# Can large language models accurately triage oculoplastic referrals?

**DOI:** 10.1038/s41433-026-04766-w

**Published:** 2026-08-07

**Authors:** Jai Paris, Ayushi Agarwal, Weng Onn Chan, Dinesh Selva

**Affiliations:** 1 Faculty of Health and Medical Sciences, Adelaide University, Adelaide, SA Australia; 2 Discipline of Ophthalmology and Vision Sciences, Adelaide University, Adelaide, SA Australia; 3 South Australian Institute of Ophthalmology, Royal Adelaide Hospital, Adelaide, SA Australia

**Keywords:** Health care, Scientific community, Medical research

Large language models (LLMs) have demonstrated promising performance across a range of medical reasoning and clinical support tasks [1, 2]. Accurate outpatient triage is essential to ensure timely access to specialist care while balancing limited health care resources [3]. Oculoplastic services encompass a broad spectrum of eyelid, lacrimal, and orbital pathologies, ranging from benign disorders to potentially sight- and life-threatening conditions. We evaluated the ability of modern LLMs to assign oculoplastic referral urgency compared with expert triage assessment.

We evaluated five widely accessible LLMs using expert oculoplastic triage as the reference standard in May 2026. One hundred hypothetical referral vignettes were developed to represent a broad range of periocular, lacrimal, and orbital conditions of varying severity encountered in routine clinical practice. Each vignette was independently reviewed by two fellowship-trained oculoplastic surgeons and assigned a triage category: Category 1 (review within 2 days), Category 2 (within 30 days), Category 3 (within 90 days), or Category 4 (within 365 days). The same referral information was then provided to ChatGPT 5.4, ChatGPT 5.5, Claude Opus 4.8 Thinking, Claude Sonnet 4.6 Thinking, and Gemini 3.1 Pro Thinking. The same concise natural language prompt was used across all models (Supplementary Material 1).

Agreement between LLM-generated and expert-assigned triage categories was assessed using exact agreement, quadratic weighted Cohen *κ*, mean absolute error (MAE), and rates of under and over-triage using SPSS (Table 1) [4]. For a secondary clinically oriented analysis, triage categories were divided into urgent (Categories 1–2) and non-urgent (Categories 3–4). Sensitivity, specificity, positive predictive value, negative predictive value, and F1 score were calculated for LLM ability to identify urgent referrals (Table 2).

**Table 1:** Performance of large language models in outpatient oculoplastic referral triage.

Model	Exact Agreement (%)	Quadratic Weighted *κ*	Mean absolute error	Under-triage rate (%)	Over-triage rate (%)
GPT-5.4	32	0.50	0.81	4	64
ChatGPT 5.5	41	0.61	0.67	8	51
Claude Opus 4.8 Thinking	37	0.58	0.72	4	59
Claude Sonnet 4.6 Thinking	31	0.49	0.82	5	64
Gemini 3.1 Pro Thinking	24	0.47	0.89	2	74

**Table 2:** Performance of large language models in identifying urgent referrals (Categories 1–2 vs 3–4).

Model	Sensitivity (%)	Specificity (%)	PPV (%)	NPV (%)	F1 Score
ChatGPT 5.5	76.1	89.7	94.8	61.9	0.85
Claude Opus 4.8 Thinking	69.0	93.1	96.1	54.0	0.81
GPT-5.4	61.9	93.1	95.7	50.0	0.75
Claude Sonnet 4.6 Thinking	60.6	93.1	95.6	42.2	0.74
Gemini 3.1 Pro Thinking	52.1	96.6	97.4	47.5	0.68

Agreement between LLM-generated and expert-assigned triage categories ranged from modest to substantial, with exact agreement rates of 24–41% and quadratic weighted *κ* values of 0.47–0.61 (Table 1). ChatGPT 5.5 demonstrated the highest concordance with expert triage (*κ *= 0.61, exact agreement 41%), followed by Claude Opus (*κ* = 0.58, exact agreement 37%). MAE ranged from 0.67–0.89 triage categories, suggesting most predictions were within 1 triage category. All models demonstrated a tendency towards assigning higher urgency categories than experts, with over-triage rates of 51–74%. Of concern, despite ChatGPT 5.5 demonstrating strong overall performance, it had the highest under-triage rate (8%).

When triage categories were split into urgent and non-urgent, sensitivity ranged from 52.1 to 76.1%, specificity from 89.7 to 96.6%, and F1 scores from 0.68 to 0.85 (Table 2). ChatGPT 5.5 achieved the highest sensitivity (76.1%) and F1 score (0.85), indicating the strongest overall performance in identifying referrals requiring expedited review. Gemini 3.1 achieved the highest specificity (96.6%) and PPV (97.4%), although this occurred alongside the lowest sensitivity (52.1%). Across all models, PPV values were consistently high (94.8–97.4%), indicating that referrals classified as urgent were usually concordant with expert triaging.

Overall, contemporary LLMs demonstrated moderate agreement with specialist oculoplastic triage decisions in hypothetical referral vignettes. Their tendency toward over-triage suggests a conservative approach that prioritises urgent pathology, although under-triage remains clinically important. Evaluation using real-world referrals is required before clinical integration.

## Supplementary information

Supplementary Material 1. Standardised Large Language Model Prompt

## Data Availability

The data that support the findings of this study are available from the corresponding author upon reasonable request.
